# Exploring the roles and therapeutic implications of melatonin-mediated KLF6 in the development of intracranial aneurysm

**DOI:** 10.1080/07853890.2024.2397568

**Published:** 2024-08-31

**Authors:** Yan Liu, Yongxing Su, Le Chen, Anzhi Li, Zhengfei Ma

**Affiliations:** Department of Neurology, Suzhou Hospital of Anhui Medical University, Suzhou, Anhui, P.R. China

**Keywords:** Intracranial aneurysm, KLF6, immune microenvironment, hypoxia, melatonin

## Abstract

**Background:**

Intracranial aneurysm (IA) is a cerebrovascular disease with a high mortality rate due to ruptured subarachnoid hemorrhage. While Krüppel-like factor 6 (KLF6) dysregulation has been implicated in cancer and cardiovascular diseases, its role in IA remains unclear.

**Materials and methods:**

The GSE122897 and GSE15629 datasets were downloaded from the Gene Expression Omnibus database. Immune cell infiltration and hypoxia analysis were performed to explore the effects of KLF6 on IA. Weighted gene co-expression network analysis was used to identify hub genes related to KLF6 expression for subsequent analyses. Hypoxia-related genes were identified. Drug prediction was performed for IA. Samples from healthy individuals and patients with IA were collected to detect the expression of endothelin-1 (ET-1), vascular hematoma factor (vWF), and KLF6. A model of H_2_O_2_-induced human brain vascular smooth muscle cells (HBVSMC) injury was constructed to explore the effects of KLF6 and melatonin to treat IA.

**Results:**

T cells CD4 memory resting and monocytes were significantly different in the KLF6 high and low expression groups. Four hypoxia-related gene sets were significantly enriched in the KLF6 high-expression group. Six hypoxia-related hub genes were obtained, which were significantly associated with KLF6. Drug prediction showed that melatonin may be a potential drug for IA. The levels of ET-1, vWF, and KLF6 were significantly upregulated in patients with IA. KLF6 exacerbates H_2_O_2_-induced injury in HBVSMC, ameliorated by melatonin.

**Conclusion:**

KLF6 may be a potential target for IA treatment, with melatonin-mediated KLF6 effects playing a crucial role in the development of IA.

## KEY MESSAGES

1. KLF6 was associated with infiltrated immune cells and hypoxia genes in intracranial aneurysm.
2. KLF6 was significantly upregulated in patients with intracranial aneurysm.
3. KLF6 exacerbates H_2_O_2_-induced injury in HBVSMC, ameliorated by melatonin.

## Introduction

Intracranial aneurysm (IA) is a common cerebrovascular disease, characterized by an abnormal protrusion of thinned arterial walls with a prevalence of 3.2% in the general population [1]. Despite the relatively low risk of rupture in patients with IA, instances of rupture lead to aneurysmal subarachnoid hemorrhage (SAH), often resulting in cerebral dysfunction or mortality [2]. Currently, the pathogenesis of IA remains unclear, and its effective treatment and prevention pose significant challenges.

The development and progression of IAs involve complex interactions between various cellular and molecular processes, including endothelial dysfunction, vascular smooth muscle cell (VSMC) changes, apoptosis, inflammation, hypoxia, and alterations in the extracellular matrix (ECM) [3,4]. Hemodynamic stress initiates the recruitment and infiltration of inflammatory cells, prompting phenotypic transformations in VSMCs in response to inflammatory injury. This cascade of events contributes to ECM changes and vascular remodeling, culminating in IA [5,6]. Furthermore, the hypoxic microenvironment fosters angiogenesis and induces inflammatory cell infiltration [7]. The crosstalk of these complex signals leads to the onset, development, and rupture of IA. Therefore, exploring the mechanisms of changes in the hypoxic and immune microenvironments in IA provides a promising avenue for the prevention and treatment of IA.

Krüppel-like factor 6 (KLF6) is a transcription factor that plays a role in various cellular processes, including cell proliferation, differentiation, and apoptosis [8]. A series of studies have shown that aberrant expression of KLF6 is implicated in the pathogenesis of a variety of diseases, such as cancer/inflammation-related diseases and cardiovascular diseases [9]. Research has revealed a link between KLF6 and matrix metalloproteinases (MMPs), enzymes crucial in ECM remodeling [10]. Furthermore, KLF6 could control inflammatory and hypoxic responses by regulating HIF1α expression in macrophages [11]. In the previous study, we demonstrated that knockdown of KLF6 alleviated H_2_O_2_-induced injury in human brain vascular smooth muscle cells (HBVSMC), suggesting that KLF6 may serve as a crucial player in the pathogenesis of IA [12]. However, the mechanism of KLF6 being involved in the development of IA remains unclear. In the present study, we aimed to investigate the potential mechanisms of KLF6 in IA and potential drugs for the treatment of IA, contributing valuable insights to the understanding and potential management of IA.

## Materials and methods

### Data sources and processing

Data for this study were downloaded from the Gene Expression Omnibus (GEO) database and processed using R3.5.3 (https://www.r-project.org/). The keyword “intracranial aneurysm” was searched in the GEO database and two datasets were included after excluding studies with single samples or those conducted at the cell line and animal levels. GSE122897 (16 controls, 21 unruptured, and 22 ruptured IA samples) and GSE15629 (5 controls, 6 unruptured, and 8 ruptured IA samples) served as the training and validation sets, respectively. Gene expression profiles were annotated using the GPL platform to convert gene probes into gene symbols, where multiple probes corresponding to the same gene are averaged.

### The characteristic analysis of KLF6

The expression levels of KLF6 between control and IA samples in GSE122897 and GSE15629 were displayed in box plots. The receiver operating signature (ROC) curves for KLF6 were analyzed using the pROC package. The area under the curve (AUC) was used to assess the diagnostic value of KLF6 for IA. Expression analysis of KLF6 in different clinical indicators subgroups was performed in the GSE122897 dataset.

### Microenvironmental analysis

IA samples were divided into high and low KLF6 expression groups based on the median expression data of KLF6 in the GSE122897 dataset. Differences in infiltration levels of immune cells between KLF6 high and low expression groups were compared using the Wilcoxon test. The immune cell composition was visualized with a bar stacked graph. Additionally, four hypoxia-related gene sets (MANALO HYPOXIA UP, MENSE HYPOXIA UP, HALLMARK HYPOXIA, and HARRIS HYPOXIA) were selected for GSEA analysis to assess the status of hypoxia.

### Gene set variation analysis (GSVA)

The “GSVA” package in R was employed to explore the differences in biological processes between high and low KLF6 expression groups. The gene set “c2.cp.kegg.v7.2.symbols” was obtained from the MSigDB database and utilized for GSVA analyses. The “limma” package in R was employed to compute the differential expression of pathways with false discovery rate (FDR) <0.05.

### Identification of differentially expressed genes (DEGs)

Multiple probes corresponding to the same gene were averaged in the GSE122897 data, and the logarithm (log_2_(data + 1)) of the processed data was taken. The “limma” package in R was used to obtain DEGs between high and low KLF6 expression groups with |log_2_FC| >1 and FDR <0.05. Volcano plots and heat maps were used to visually represent the analysis results.

### Weighted gene co-expression network analysis (WGCNA)

A scale-free gene co-expression network was constructed using the “WGCNA” package in R. First, 43 IA sample data were clustered using the average linkage method in “hclust” function to detect outliers. We chose 70 as the cut tree height to remove outliers. To construct the scale-free topology, the “pickSoftThreshold” function was performed (β = 6). The height was set to 0.90, and the minimum number of genes in the module was set to 150. Based on the kernel value, the adjacency matrix was transformed into the topological overlap matrix (TOM) and the corresponding dissimilarity matrix (1-TOM). Using default parameters, the “cutreeDynamic” function was used to divide modules based on genes with similar expression patterns. A truncation of 0.25 height was applied to the modules identified by the dynamic tree-cutting algorithm since they may be similar. Phenotypic data representing the KLF6 high and low expression groups were extracted and analyzed for their association with WGCNA modules. Ultimately, hub modules related to high and low KLF6 expression groups were selected.

### Hub gene and enrichment analysis

Hub genes were identified based on the module connectivity and correlation with high and low KLF6 expression groups obtained from WGCNA analysis. Module connectivity was defined as the absolute value of the Pearson correlation between genes (MM). The relationship between genes and high/low KLF6 expression groups was defined as the absolute value of the Pearson correlation between each gene and the high/low KLF6 expression groups (GS). The genes with |MM| >0.6 and |GS| >0.4 in the hub module were defined as high and low KLF6 expression groups related genes. The genes in the hub module and DEGs were overlapping to obtain hub genes. Gene ontology (GO) enrichment analysis was performed to investigate the function of hub genes using the DAVID database (https://david.ncifcrf.gov/) with *p* < 0.01.

### Hypoxia analysis

Hypoxia-related genes were obtained from the MSigDB database (https://www.gsea-msigdb.org/gsea/msigdb/) and overlapped with hub genes to obtain hypoxia-related hub genes, followed by correlation analysis.

### Signature selection and model building

Machine learning algorithm modeling was used to identify biomarkers for the diagnosis of IA patients with different KLF6 expressions. Firstly, the signature genes were identified from hub genes using the least absolute shrinkage and selection operator (LASSO) regression analysis with “glmnet” package in R. Secondly, the “randomForest” package in R was used to rank the importance of signature genes selected by LASSO in descending order according to the value of the average decreasing precision. The optimal number of features was obtained by adding one gene at a time using the random forest algorithm and a top-down approach with 10-fold cross-validation. Finally, random forest (“randomForest”) and support vector machine (“e1071”) classification models were built. The diagnostic ability of models and biomarkers was evaluated using ROC curves.

### Drug prediction

Differential expression analysis was conducted on hub genes between IA samples and controls. Subsequently, the DGIdb (https://dgidb.org/) database was used to find differential hub gene-related drugs.

### Cell culture and protocols

HBVSMC (Cat. No. CM-H116) were purchased from Procell (Wuhan, China) and cultured using DMEM medium containing 10% fetal bovine serum (FBS) at 37 °C with 5% CO_2_. The trypsin-digested cell suspension was seeded into 6-well plates (2 × 10^5^/well). The adherent cells were divided into six groups: Control group, H_2_O_2_ group, H_2_O_2_+Melatonin, H_2_O_2_+pcDNA-NC group, H_2_O_2_+pcDNA-KLF6 group, and H_2_O_2_+pcDNA-KLF6 + Melatonin group. Except the control group, the other groups were treated with 100 μM H_2_O_2_ for 6 h, while the control group received an equivalent volume of PBS. Transfections of pcDNA-KLF6 and pcDNA vectors were conducted using Lipofectamine 3000 (Thermo Scientific, USA) according to the manufacturer’s instructions. Additionally, in the H_2_O_2_+Melatonin and H_2_O_2_+pcDNA-KLF6 + Melatonin group, 100 μM melatonin was administered for 6 h in addition to the above treatments.

### Real-time quantitative PCR (RT-qPCR)

Total RNA was extracted from cells using TRIzon Reagent (cwbio, China) and reversed into cDNA using Evo M-MLV Reverse Transcription Reagent (Accurate Biology, China). The expression levels of KLF6 were detected with RT-qPCR, which used GADPH as an internal reference gene. qPCR was performed using the SYBR Green Pro Taq HS Mix (Takara Bio, Japan). The reaction system was programmed as follows: incubation at 95 °C for 30 s, followed by 40 cycles for 5 s at 95 °C, and 30 s at 60 °C for three times. The relative expression was determined using the 2^-ΔΔct^ method. *p* < 0.05 was considered statistically significant. Primer sequences were manifested in the following: KLF6: forward primer: 5′-GGCCAAGTTTACCTCCGACC-3′; reverse primer: 5′-TAAGGCTTTTCTCCTTCCCTGG-3′. GAPDH: forward primer: 5′-TTCTTTTGCGTCGCCAGGTG-3′; reverse primer: 5′-GGAGGGAGAGAACAGTGAGC-3′.

### Cell proliferation assays

3-(4,5-dimethylthiazol-2-yl)-2,5-diphenyltetrazolium bromide (MTT), plate clone and 5-ethynyl-2′-deoxyuridine (EDU) were used to evaluated cell proliferation. Cells used for MTT assay were seeded in the 96-well plate (6000/well) and cultured for 24, 48, 72, and 96 h. Then, 20 μL of 5 mg/mL MTT (Sangon Biotech, China) was added for 4 h, the culture medium was completely aspirated, and 100 μL DMSO was added. The OD value was detected at 490-570 nm using enzyme labeling equipment (Thermo Fisher, USA). Cells used for plate clone were seeded in the 6-well plate (1000/well) and cultured until 11 days, during which the medium was changed every 3 days. Subsequently, cells were fixed with 1 mL of 4% paraformaldehyde (Sinopharm Chemical Reagent Co., Ltd, China) and incubated with crystal violet staining solution (Sangon Biotech). The cell clones were photographed under a fluorescence microscope (Olympus, Japan). Click-iT EdU-488/555/647 Cell Proliferation Assay Kits (Servicebio, China) were used to perform EDU assay according to the manufacturer’s instructions, respectively.

### Western blot

The protein separation was performed through 10% polyacrylamide gel, electroblotting was performed onto a polyvinylidene fluoride membrane, and then blocking with 5% skim milk powder was implemented. After incubation with KLF6 (1:800), GAPDH (1:50000), Bax (1:20000, Proteintech, USA), Bcl-2 (1:2000), cleaved caspase-3 (1:1000, Abcam, UK) at 4 °C overnight, secondary antibody labeled with horseradish peroxidase (1:5000, ZSGB-Bio, China) were added, final incubated with ECL was for quantification (Servicebio), and Image J software was applied for quantitative analysis.

### Flow cytometry

Cell apoptosis was evaluated using Annexin V-PE/7-AAD Apoptosis Assay Kit (Meilunebio, China) according to the manufacturer’s instructions. Three control groups were established: normal cells, cells with 7-AAD staining, and cells with Annexin V-PE staining. The results were analyzed using CellQuest software, and two-color dot plots were generated with PE on the x-axis and 7-AAD on the y-axis. The rate of apoptosis was calculated as the total number of Annexin V-PE^+^ 7-AAD^+^ cells and Annexin V-PE^+^ cells.

### Patient samples and protocols

A total of 28 samples were collected from 12 healthy controls and 16 patients with IA as previously described [12]. The mRNA and protein levels of KLF6 in blood were detected by RT-qPCR and western bot, respectively. The levels of vascular hematoma factor (vWF) and endothelin-1 (ET-1) in serum were detected using Enzyme-Linked Immunosorbent Assay (ELISA) Kit (Shanghai Fanke, China) according to the instructions. The approval of the research was obtained through the Medical Ethics Committee of the Suzhou Hospital of Anhui Medical University (C2024002), following the principles of the Declaration of Helsinki. Written informed consent was obtained from the participants in the investigation.

### Statistical analysis

The expression of genes and levels of immune infiltration in different groups were compared using the Wilcoxon test. The correlation between immune cells and KLF6 was calculated using the Pearson correlation coefficient method. SPSS 22.0 was used for statistical analysis of the data and the expression of the results in experiments was presented as mean ± standard deviation. An independent sample t-test and an ANOVA for variance analyzed sample comparisons between two and multiple groups. *p* < 0.05 was considered a statistically significant difference.

## Results

### The characteristic analysis of KLF6

**Figure 1.**
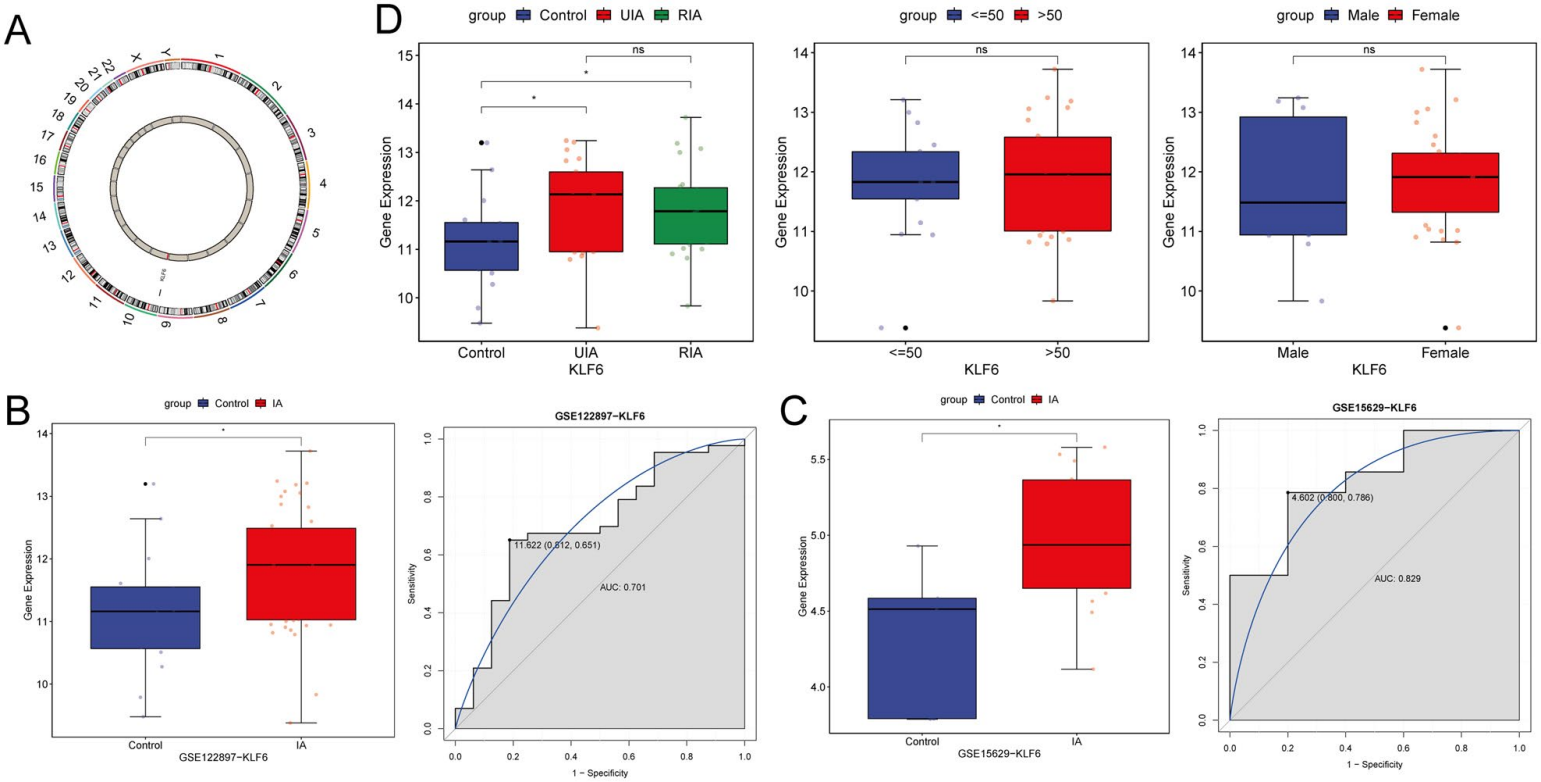
The characteristic analysis of KLF6. (A) Location of KLF6 on the chromosome. (B-C) The expression levels of KLF6 and ROC analysis in datasets GSE122897 and GSE15629. (D) The expression levels of KLF6 in different clinical indicators, including ruptured and unruptured conditions, age, and gender. UIA/RIA, unruptured/ruptured intracranial aneurysm.

The KLF6 gene is located on chromosome 10 (Figure 1A). In datasets GSE122897 and GSE15629, KLF6 exhibited significantly higher expression levels in IA samples compared with controls, with AUC values of 0.701 and 0.829, respectively (Figures 1B and 1C). These AUC values indicated that KLF6 had a better diagnostic potential for IA. Furthermore, there was no difference of KLF6 expression between patients with different clinical indicators in the GSE122897 dataset, including ruptured and unruptured IA, as well as different age groups and genders (Figure 1D).

### Microenvironment and KLF6

**Figure 2.**
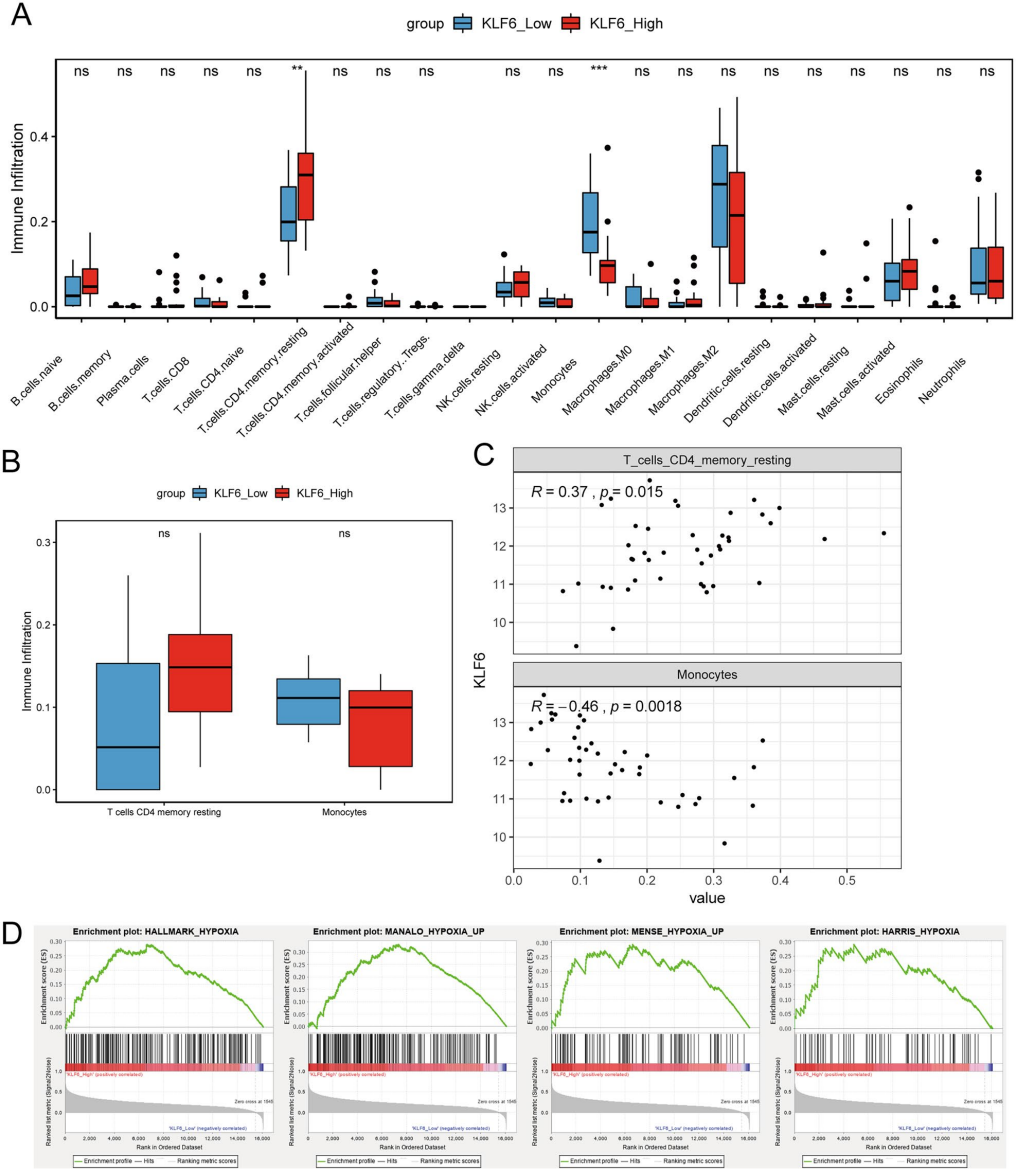
Microenvironmental analysis of IA. (A) The infiltration levels of different immune cells in high and low KLF6 expression groups. (B) The infiltration levels of T cells CD4 memory resting and monocytes in the GSE15629 dataset. (C) Correlation analysis of T cells CD4 memory resting and monocytes with KLF6. (D) GSEA analysis of the hypoxia gene sets in high and low KLF6 expression groups.

Based on the KLF6 expression data in the GSE122897 dataset, IA samples were divided into high and low KLF6 expression groups. In the dataset GSE122897, there were 22 samples in the low KLF6 expression group, and 21 samples in the high KLF6 expression group. Microenvironmental analysis revealed significant differences in T cells CD4 memory resting and monocytes between these two groups (Figure 2A). While the GSE15629 dataset did not show statistically significant differences in T cells CD4 memory resting and monocytes between the high and low KLF6 expression groups, there was a similar trend observed (Figure 2B). Furthermore, correlation analysis revealed a significant association between these two immune cell types and KLF6. Monocytes and KLF6 were negatively correlated (r = −0.46), while T cells CD4 memory resting and KLF6 were positively correlated (*r* = 0.37) (Figure 2C). Additionally, the relationship between hypoxia and KLF6 was investigated using GSEA, revealing that all four hypoxia-associated gene sets were enriched in the high KLF6 expression group. Notably, MANALO HYPOXIA UP and HALLMARK HYPOXIA showed significant enrichment in the high KLF6 expression group (*p* < 0.05) (Figure 2D). Collectively, these findings suggested KLF6 may be associated with immune microenvironment and hypoxia in IA.

### GSVA analysis

**Figure 3.**
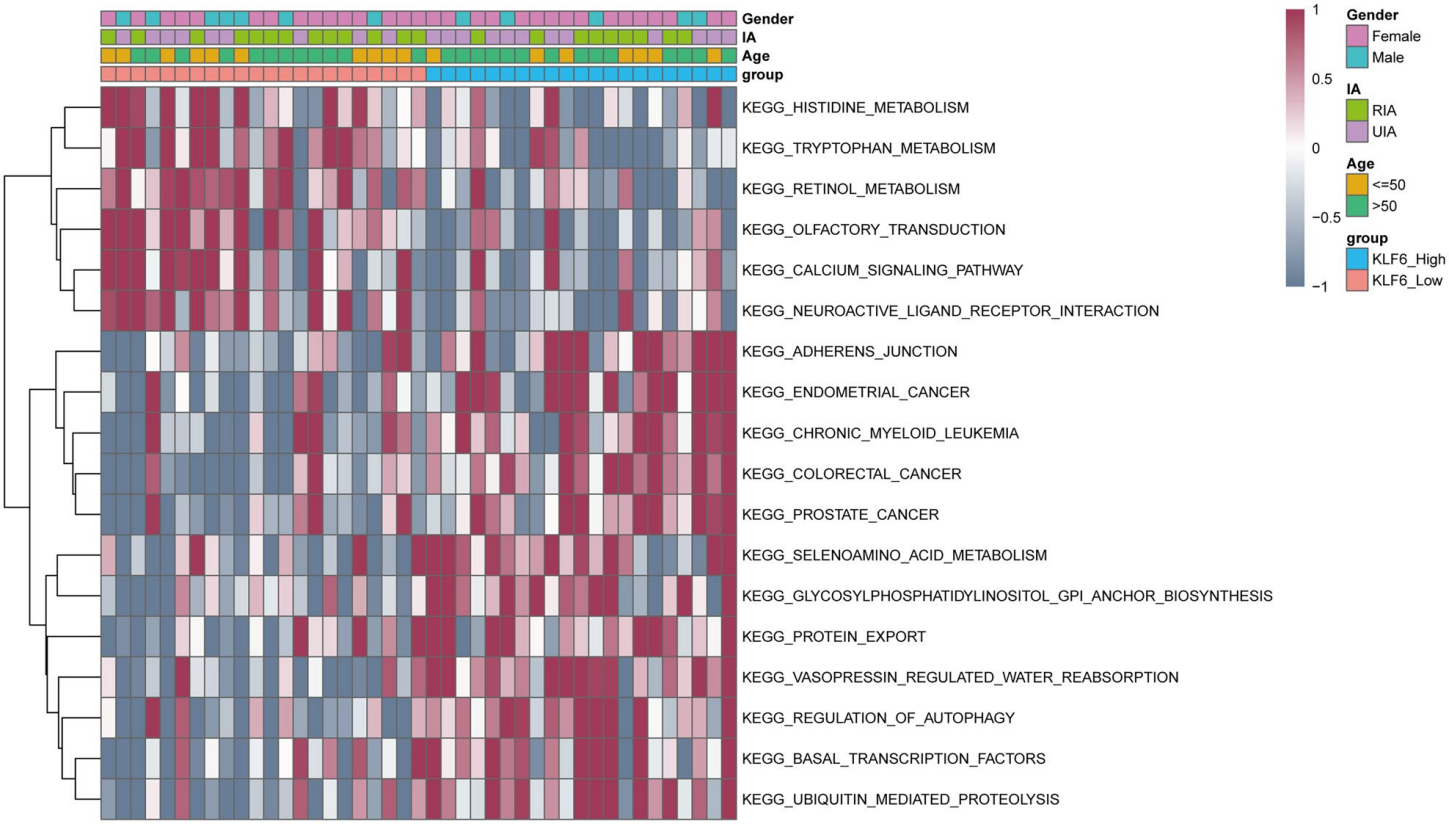
GSVA analysis. GSVA analysis in high and low KLF6 expression groups.

To investigate the differences in biological processes between high and low KLF6 expression groups, we performed the GSVA. The results showed that 18 pathways were screened out, in which 6 pathways were more active in the KLF6 low expression group, such as neuroactive ligand-receptor interaction, tryptophan metabolism, histidine metabolism, retinol metabolism, etc.; 12 pathways were more active in the KLF6 high-expression group, such as regulation of autophagy, ubiquitin mediated proteolysis, protein export, basal transcription factors, glycosylphosphatidylinositol (GPI) anchor biosynthesis (Figure 3). These results suggested that the expression level of KLF6 could potentially affect multiple biological processes in IA.

### Identification of DEGs and hub genes

**Figure 4.**
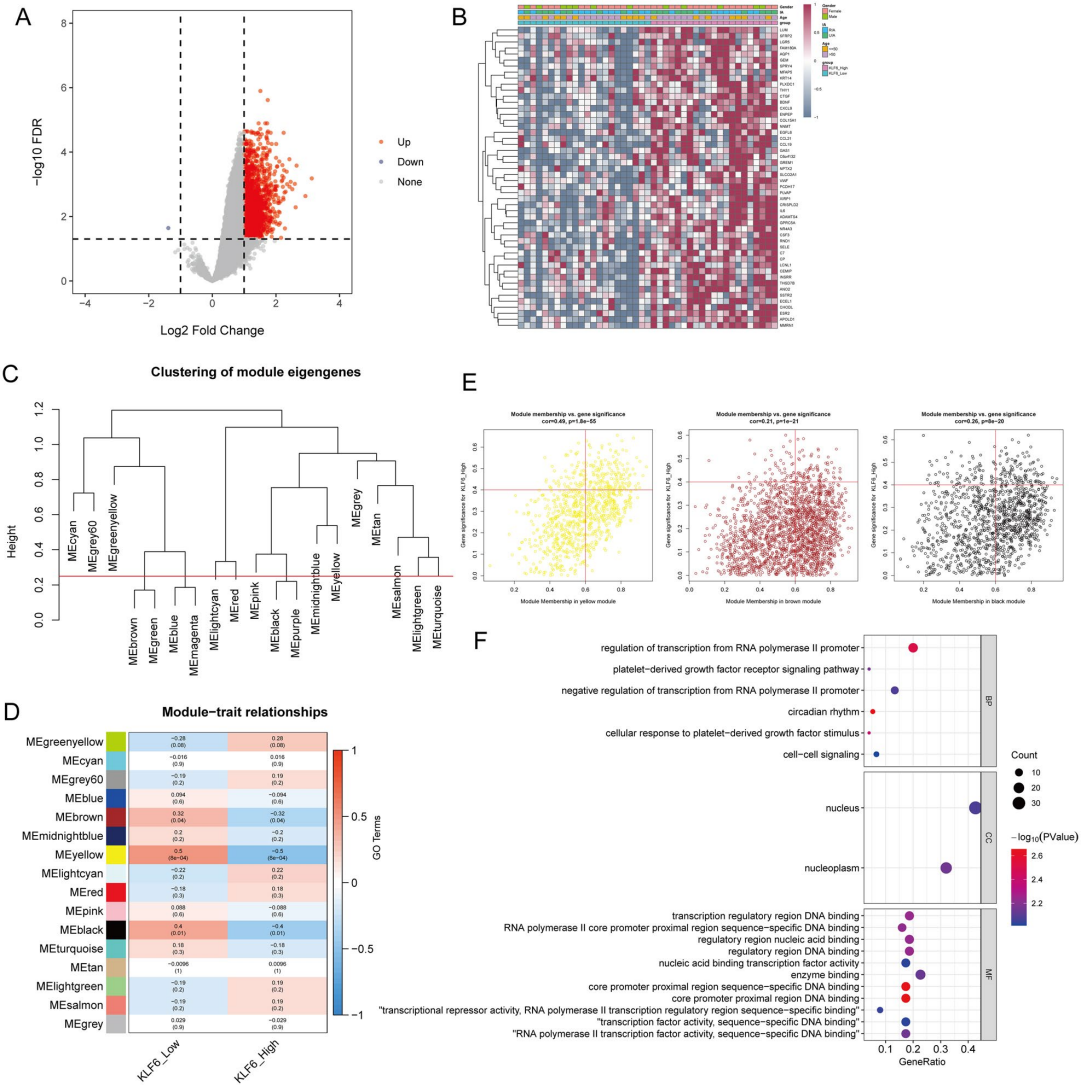
Identification of DEGs and hub genes. Identification of DEGs in high and low KLF6 expression groups and presented as (A) a volcano and (B) heat maps. (C) Clustering of module eigengenes. (D) Heatmap of the correlation between the module eigengenes and KLF6 expression. MEyellow, MEbrown, and MEblack modules were selected for subsequent analysis. (E) The scatter plots to show the correlation for MM (X-axis) and GS (Y-axis) in three modules. (F) GO enrichment analysis of hub genes.

Compared with the low KLF6 expression group, 1840 DEGs were identified in the high KLF6 expression group, including 1839 up-regulated DEGs and 1 down-regulated DEGs (Figure 4A and 4B). Sixteen candidate modules were screened by WGCNA (Figure 4C). Among them, three modules were selected as being significantly associated with the KLF6 high and low expression groups, including MEbrown (*p* = 0.04), MEyellow (*P* = 8e-04), and MEblack (*p* = 0.01) (Figure 4D), and these three modules were used as hub modules. In these hub modules, a total of 396 genes were found to be associated with the high and low KLF6 expression groups (Figure 4E). These 396 genes and DEGs were overlapped to obtain 76 hub genes. Subsequent enrichment analysis showed that these hub genes were mainly localized in the nucleoplasm and nucleus, etc. Additionally, they may play key roles in transcriptional regulation, participating in biological processes such as circadian rhythms, RNA polymerase II transcriptional regulation, platelet-derived growth factor (PDGF) signaling pathway, and cell signaling (Figure 4F).

### Hub genes and hypoxia

**Figure 5.**
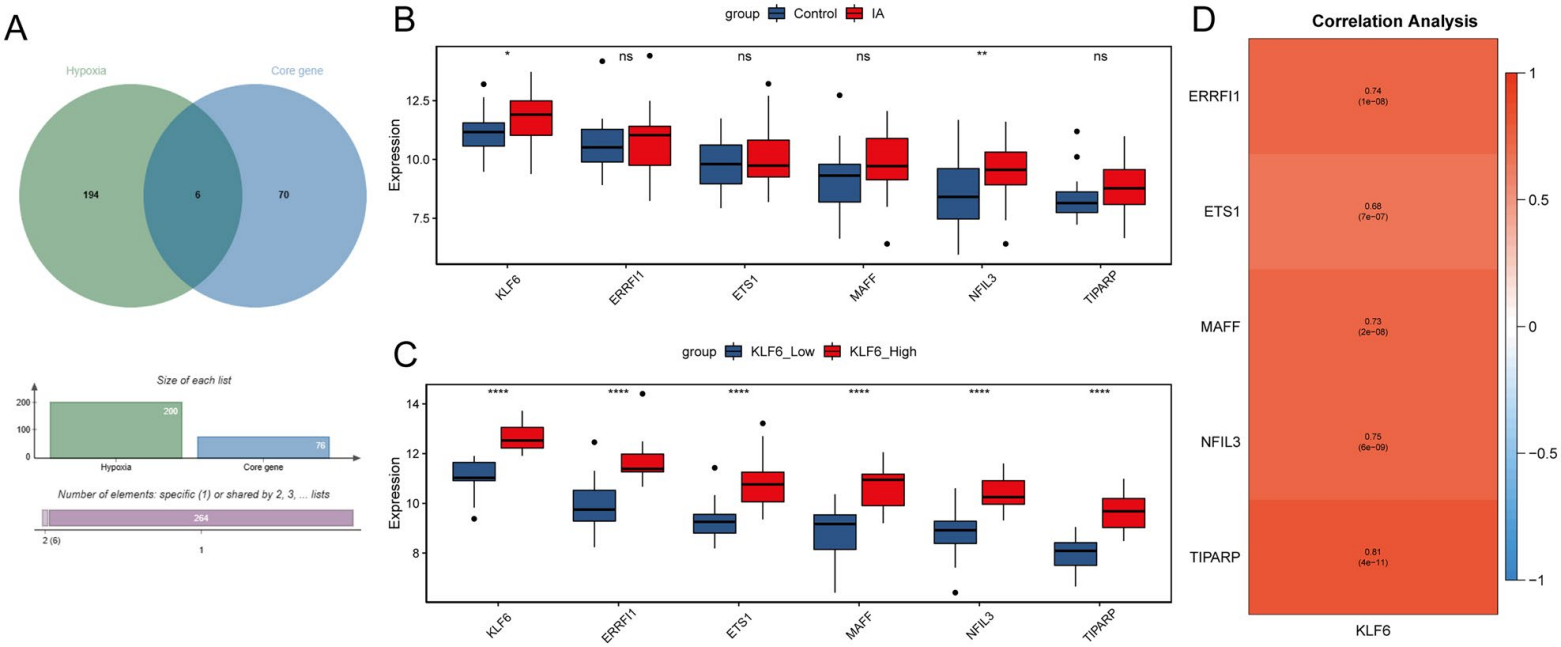
Hypoxia analysis. (A) Hypoxia-related genes and hub genes were overlapped to obtain hypoxia-related hub genes. (B) The expression levels of hypoxia-related hub genes between IA and control samples. (C) The expression levels of hypoxia-related hub genes between high and low KLF6 expression groups. (D) Correlation analysis of KLF6 with hypoxia-related hub genes.

We further explored the relationship between KLF6 and hypoxia. A total of 200 hypoxia-related genes were obtained from the MSigDB database. These hypoxia genes were then overlapped with hub genes to identify hypoxia-related hub genes (KLF6, ERRFI1, ETS1, MAFF, NFIL3, and TIPARP) (Figure 5A). Among them, KLF6 and NFIL3 were significantly highly expressed in IA compared with controls, while ERRFI1, MAFF, and TIPARP had a tendency to be highly expressed (Figure 5B). Furthermore, ERRFI1, ETS1, MAFF, NFIL3, and TIPARP were significantly highly expressed in the high KLF6 expression group and positively correlated with KLF6, with the strongest correlation observed between TIPARP and KLF6 (*r* = 0.81) (Figure 5C and 5D).

### Identification of signature genes and models

**Figure 6.**
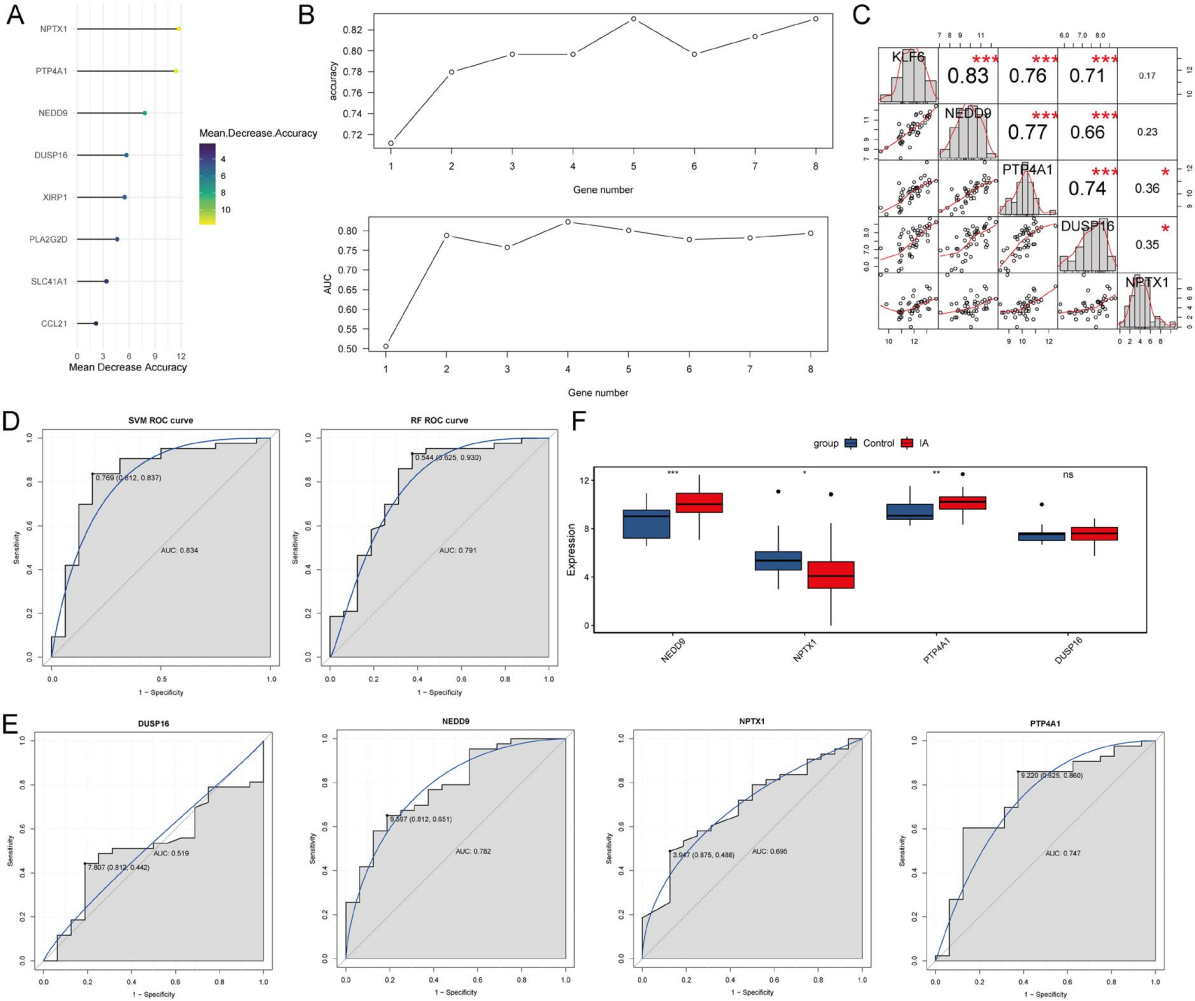
Identification of signature genes and construction of diagnostic models. (A) Eight genes were screened using LASSO analysis and ranked by mean decrease accuracy. (B) 10-fold cross validation of 8 genes to select optimal gene number. NPTX1, PTP4A1, NEDD9, and DUSP16 were selected as signature genes for subsequent analysis. (C) Correlation analysis of KLF6 with signature genes. (D) ROC analysis of SVM and RF. (E) ROC analysis of individual signature gene. (F) The expressions of signature genes between IA and control samples.

Eight genes were identified from 76 hub genes using LASSO analysis and ranked by mean decrease accuracy (Figure 6A). The results of the 10-fold cross-validation showed that the highest accuracy was achieved when the number of genes was four (Figure 6B). Therefore, four genes were selected as the signature genes, including NPTX1, PTP4A1, NEDD8, and DUSP1. Correlation analysis showed that NEDD9, PTP4A1, and DUSP16 were strongly correlated with KLF6 (Figure 6C). Classification models were then constructed using four signature genes and ROC analysis showed that the AUCs for SVM and RF were 0.834 and 0.791, respectively (Figure 6D). Importantly, the AUCs of the SVM and RF models exceeded the AUC of the individual signature gene (Figure 6E), emphasizing their collective diagnostic efficacy. Furthermore, the expression levels of NEDD9, NPTX1, and PTP4A1 showed significant differences between IA samples and controls (Figure 6F). In addition, these results were consistent with those validated in dataset GSE15629 (Figure S1).

### Drug prediction

**Figure 7.**
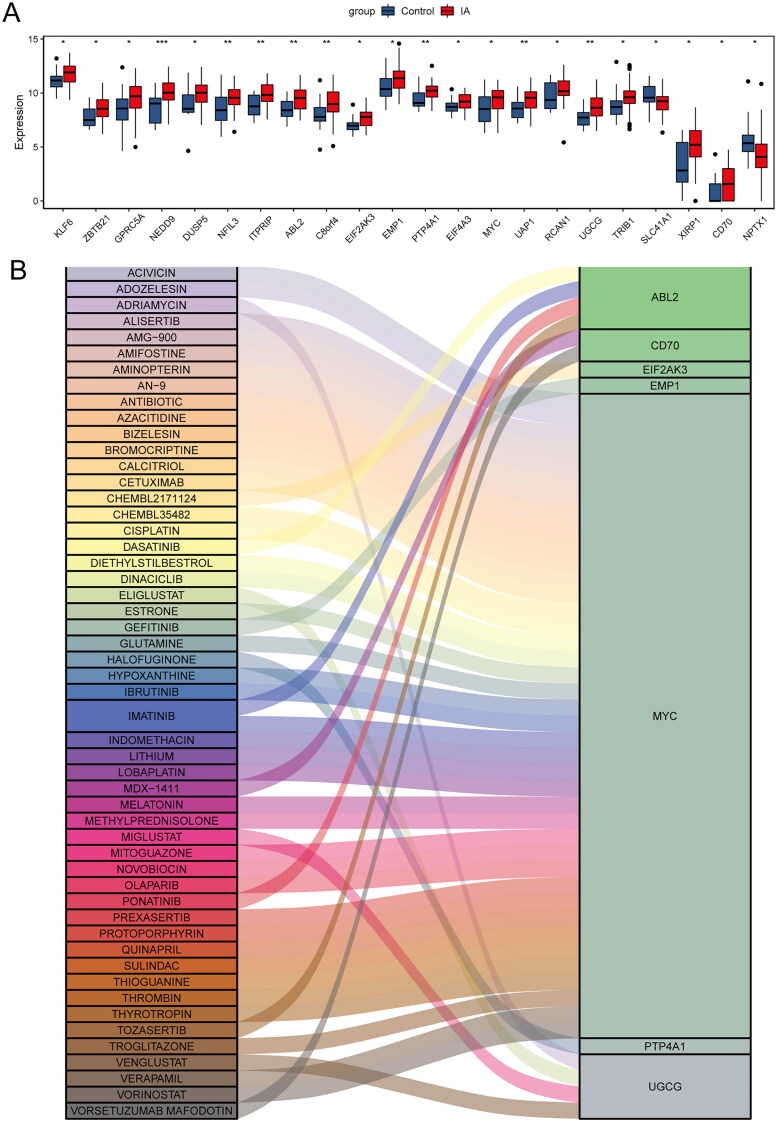
Drug prediction. (A) Differential expression analysis of hub genes in IA and control samples. (B) Drugs-genes interactions in the DGldb database.

Differential expression analysis of 76 hub genes revealed a total of 22 hub genes that were significantly different between control and IA samples (Figure 7A). Among these 22 hub genes, 7 hub genes were found to be associated with drugs using the DGIdb database. The highest number of drugs were associated with MYC, such as melatonin (Figure 7B).

### KLF6 was upregulated in patients with IA

**Figure 8.**
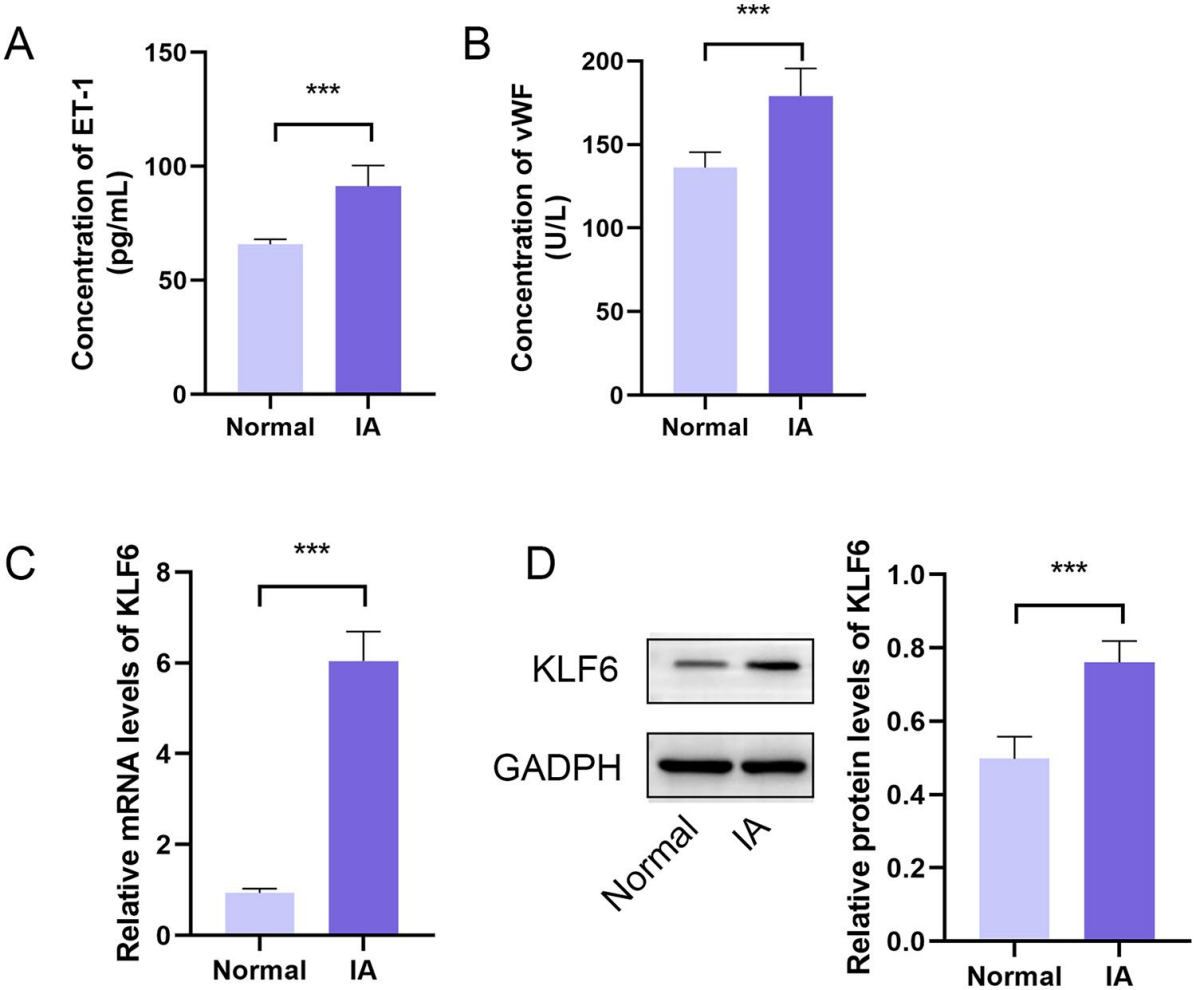
The levels of ET-1, vWF, and KLF6 in IA. (A) The levels of ET-1 and (B) vWF in serum were detected by ELISA. (C) The mRNA and (D) protein levels of KLF6 in blood were detected by RT-qPCR and Western blot, respectively. *p < 0.05, **p < 0.01, ***p < 0.001.

ET-1 and vWF are typically associated with vascular wall damage and inflammatory processes. The levels of ET-1 and vWF were significantly increased in patients with IA (Figure 8A and 8B). Further, the mRNA and protein levels of KLF6 were significantly increased in patients with IA (Figure 8C and 8D).

### Overexpressed KLF6 exacerbated H_2_O_2_-induced HBVSMC injury

**Figure 9.**
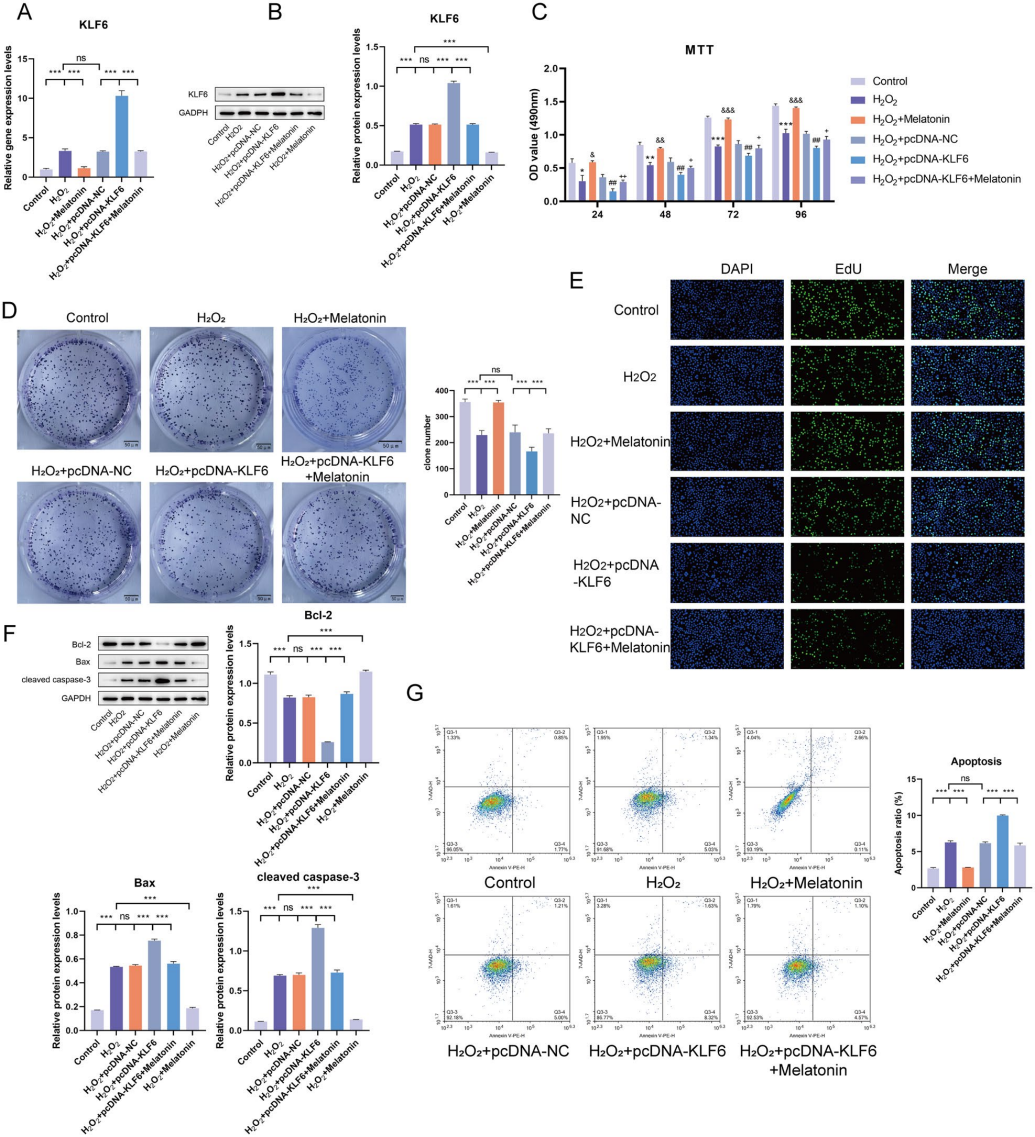
Melatonin alleviates the exacerbation of H_2_O_2_-induced HBVSMC injury by KLF6. (A) The mRNA and (B) protein levels of KLF6 were detected using RT-qPCR and Western blot. (C-E) MTT, plate cloning, and EDU were performed to detect HBVSMC cell proliferation. * vs. H_2_O_2_, & vs. H_2_O_2_, # vs. H_2_O_2_+pcDNA-NC, + vs. H_2_O_2_+pcDNA-KLF6 + melatonin. (F) The protein levels of apoptosis-related factors Bcl-2, bax, and cleaved caspase-3. (G) Flow cytometry was performed to detect HBVSMC cell apoptosis. ***p < 0.001.

H_2_O_2_-induced HBVSMC injury model was established to simulate the progression of IA. The results showed that KLF6 expression was significantly increased in the H_2_O_2_ group compared with controls (Figure 9A and 9B). The upregulation of KLF6 expression in IA patients prompted us to consider that KLF6 could promote IA progression. Therefore, an overexpression vector for KLF6 was utilized before H_2_O_2_ stimulation of HBVSMC to explore the effect of KLF6 on HBVSMC injury. MTT results indicated a substantial reduction in cell proliferation in the H_2_O_2_-induced model compared with controls (Figure 9C). Upon overexpression of KLF6, cell proliferation further decreased. These results were consistently validated through plate cloning and EDU assay, which were in agreement with the MTT findings (Figure 9D and 9E). The protein levels of pro-apoptotic factors Bax and cleaved caspase3 were significantly increased, while anti-apoptotic factor Bcl-2 was significantly decreased in the H_2_O_2_-induced model, indicating increased apoptosis (Figure 9F). Upon overexpressing KLF6, cell apoptosis further increased. The flow cytometry results were consistent with and supported these observations (Figure 9G). Taken together, KLF6 could exacerbate H_2_O_2_-induced injury in HBVSMC.

### Melatonin alleviates H_2_O_2_-induced HBVSMC injury by modulating KLF6

We further investigate the roles of melatonin in H_2_O_2_-induced model. The results suggested melatonin treatment reduced the levels of KLF6 in the model (Figure 9A and 9B). When comparing the H_2_O_2_ group with the H_2_O_2_+melatonin group, it was observed that melatonin enhanced cell proliferation and reduced apoptosis (Figure 9C–9G). These observations suggested a potential trend in these measures toward normal levels. Moreover, compared to the H_2_O_2_+pcDNA-KLF6 group, there was an increase in cell proliferation and a decrease in apoptosis in the H_2_O_2_+pcDNA-KLF6 + melatonin group (Figure 9C–9G), suggesting that melatonin could alleviate the exacerbation of H_2_O_2_-induced HBVSMC injury by KLF6. Therefore, our results indicated that melatonin could alleviate H_2_O_2_-induced HBVSMC injury by modulating KLF6.

## Discussion

IAs are known as cerebral aneurysms, a relatively common condition with a high mortality rate after rupture if left untreated. In fact, 12% of patients with ruptured IA-induced SAH die immediately [13]. Despite ongoing research, the mechanisms of occurrence, progression, and rupture in IA remain unclear. Hypoxia and the immune microenvironment have been reported to play a role in the pathogenesis of IA [14]. In this study, we systematically analyzed the potential role of KLF6 in IA, explored its effects on hypoxia and the immune microenvironment, and identified potential therapeutic drugs for IA.

KLF6 belongs to the Sp1/KLF family of transcription factors, which can regulate gene transcription and its aberrant expression is associated with a variety of diseases [15,16]. In our study, KLF6 expression was significantly higher in IA than in normal samples. ROC analysis demonstrated the diagnostic potential of KLF6 for IA. GO analysis showed that 76 hub genes were enriched in transcriptional regulation, cell signaling and PDGF signaling pathways. Previous studies have shown that these biological processes play critical roles in maintaining vessel wall structure and function, and their abnormalities may affect IA formation and progression [17–20]. Taken together, our study indicated that KLF6 may play a key role in the pathogenesis of IA and hold promise as a biomarker.

Critical to our understanding is the correlation between KLF6 and immune cell dynamics in IA. Hemodynamic stress triggers the pathogenesis of IA, leading to excessive inflammatory and immune responses in the vessel wall [21]. Immune cell infiltration is a crucial process in the development of IA [22]. Our study suggested KLF6 may influence the infiltration of T cells CD4 memory resting and monocytes, which are known to be dysregulated in patients with IA [23] and contribute to the inflammatory milieu in IA [24,25]. Previous studies showed that KLF6 is involved in endothelial dysfunction and regulates macrophage-mediated inflammation [26,27], providing a link between KLF6 and immune cells in IA.

Several studies have demonstrated the close relationship between hypoxia and the pathogenesis of IA. Meanwhile, KLF6 was significantly upregulated in ischemia-reperfusion injury, a pathological process closely related to hypoxia, and was associated with post-injury inflammation and apoptosis [28]. In this study, GSEA results showed that hypoxia-related gene sets were enriched in the high KLF6 expression group. Additionally, our study identified six hypoxia-related hub genes significantly associated with KLF6, which could influence rupture in IA [29] or angiogenesis in cancer [30,31], further supporting the possibility of KLF6 influencing the hypoxic microenvironment in IA.

The classification model constructed based on the four genes (NPTX1, PTP4A1, NEDD8, and DUSP16) has a good diagnostic ability for precise typing and treatment choices of IA patients to achieve individualized medicine. Studies showed that these signature genes play important roles in neural-related angiogenesis [32], cell signaling [33], protein ubiquitination [34], and cell cycle regulation [35], respectively. These were generally consistent with the GSVA results in the high and low KLF6 expression groups.

VSMCs play a key role in the pathogenesis of IA [36]. Oxidative stress-induced inflammatory response leads to phenotypic modulation of VSMCs and involves dysregulation of ECM remodeling and VMSC apoptosis, which further contributes to increased risk of arterial wall thinning and rupture [37,38]. It was shown that KLF6 translocation leads to the release of IL-6 from VSMCs, and is involved in the mechanisms of vascular injury and vascular remodeling [39]. In addition, KLF6 is closely related to the TGF-β pathway in endothelial cell vascular remodeling [40]. Our results showed that KLF6 overexpression exacerbated the H_2_O_2_-induced HBVSMC injury. Therefore, KLF6 could be a possible therapeutic target for IA.

Melatonin, extensively studied for its antioxidant, pro-inflammatory, and anti-inflammatory effects [41], significantly inhibits the NF-κB signaling pathway and the activity of MMPs, making it a potential therapeutic option for the treatment of abdominal aortic aneurysms in rats [42]. Melatonin has been shown to reduce SAH-associated inflammation and high levels of melatonin in serum are associated with poor prognosis in patients with SAH [43,44]. Melatonin could regulate pro-inflammatory cytokines and immune cells [45], which are closely related to the immune microenvironment. In addition, melatonin is associated with hypoxia and may ameliorate hypoxia-associated injury [46]. Our results revealed that melatonin alleviates the exacerbation of H_2_O_2_-induced HBVSMC injury by KLF6, suggesting the potential therapeutic value of melatonin for IA.

This study has some limitations. Firstly, due to patient heterogeneity, larger cohorts with alternative analytical methods are necessary to confirm the robustness of the results. Secondly, the potential mechanisms of KLF6 in hypoxia and the immune microenvironment of IA were derived from bioinformatics analyses and need to be validated through additional functional experiments. Lastly, although our study demonstrated the therapeutic potential of melatonin in an *in vitro* model of IA, further experiments are needed to validate the efficacy and safety of melatonin. These validations will provide further insights for therapeutic strategies in clinical practice.

In conclusion, KLF6 may play crucial roles in the onset and progression of IA, affecting hypoxia and the immune microenvironment. Furthermore, KLF6 is probably a target for the treatment, and melatonin mediated KLF6 effects is important in the development of IA. These findings are important for understanding the pathogenesis, diagnosis, and treatment of IA.

## Supplementary Material

Supplemental Material

## Data Availability

The datasets used and/or analysed during the current study are available from the corresponding author on reasonable request.
