# Supplementary material for: Exploring the roles and therapeutic implications of melatonin-mediated KLF6 in the development of intracranial aneurysm
Source: Ann Med. 2024 Aug 31;56(1):2397568. doi: 10.1080/07853890.2024.2397568 (PMC11370671; doi:10.1080/07853890.2024.2397568)

Figure S1. Validation of signature genes and diagnostic models in GSE15629. (A) ROC analysis of SVM and RF. (B) ROC analysis of individual signature gene. (C) The expressions of signature genes between IA and control samples


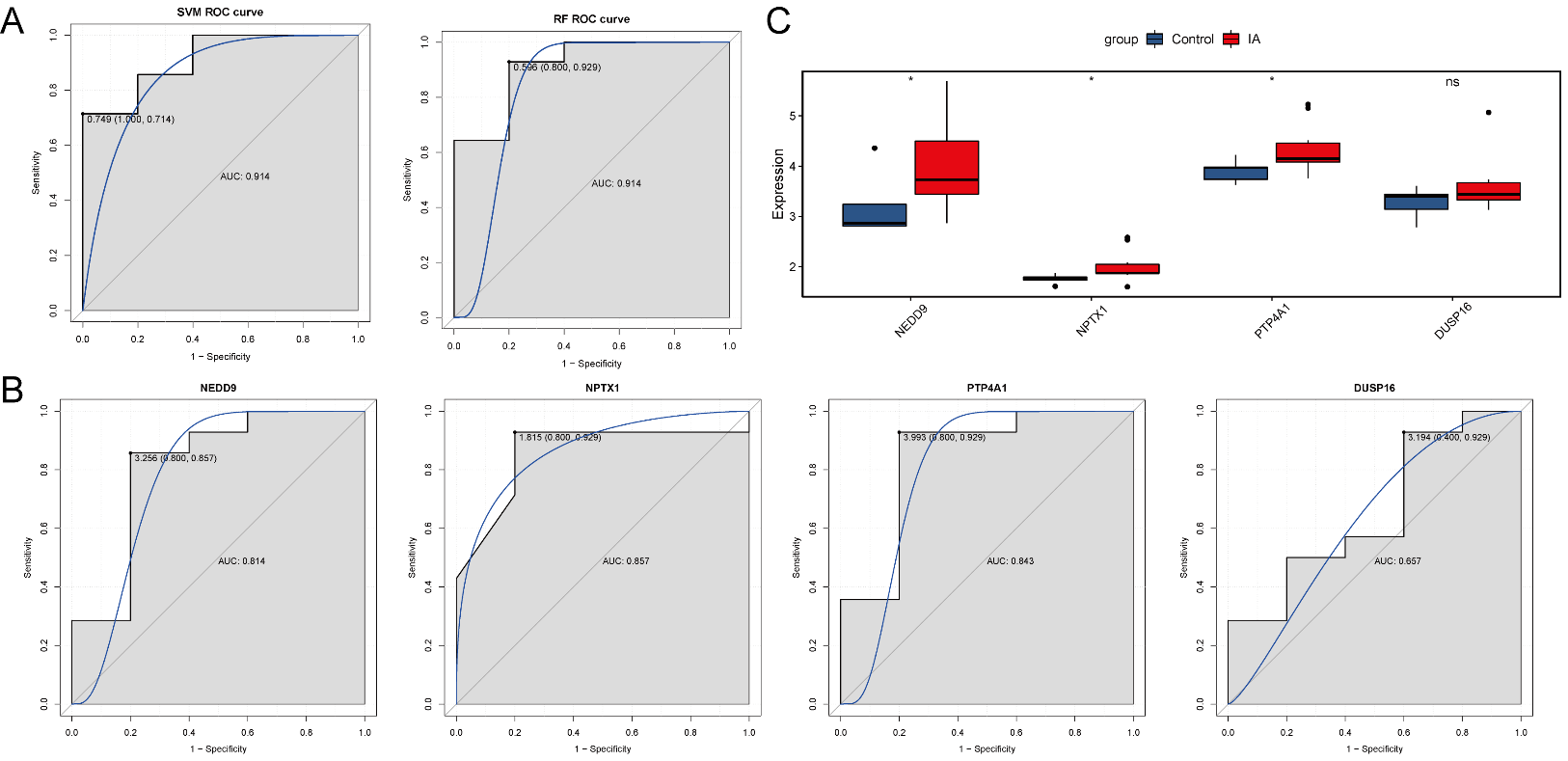

Supplement: Supplemental Material [file IANN_A_2397568_SM2661.zip › suppl_data/Figure S1.docx]
