# Supplementary figures and images for: Exploring the roles and therapeutic implications of melatonin-mediated KLF6 in the development of intracranial aneurysm
Source: Ann Med. 2024 Aug 31;56(1):2397568. doi: 10.1080/07853890.2024.2397568 (PMC11370671; doi:10.1080/07853890.2024.2397568)

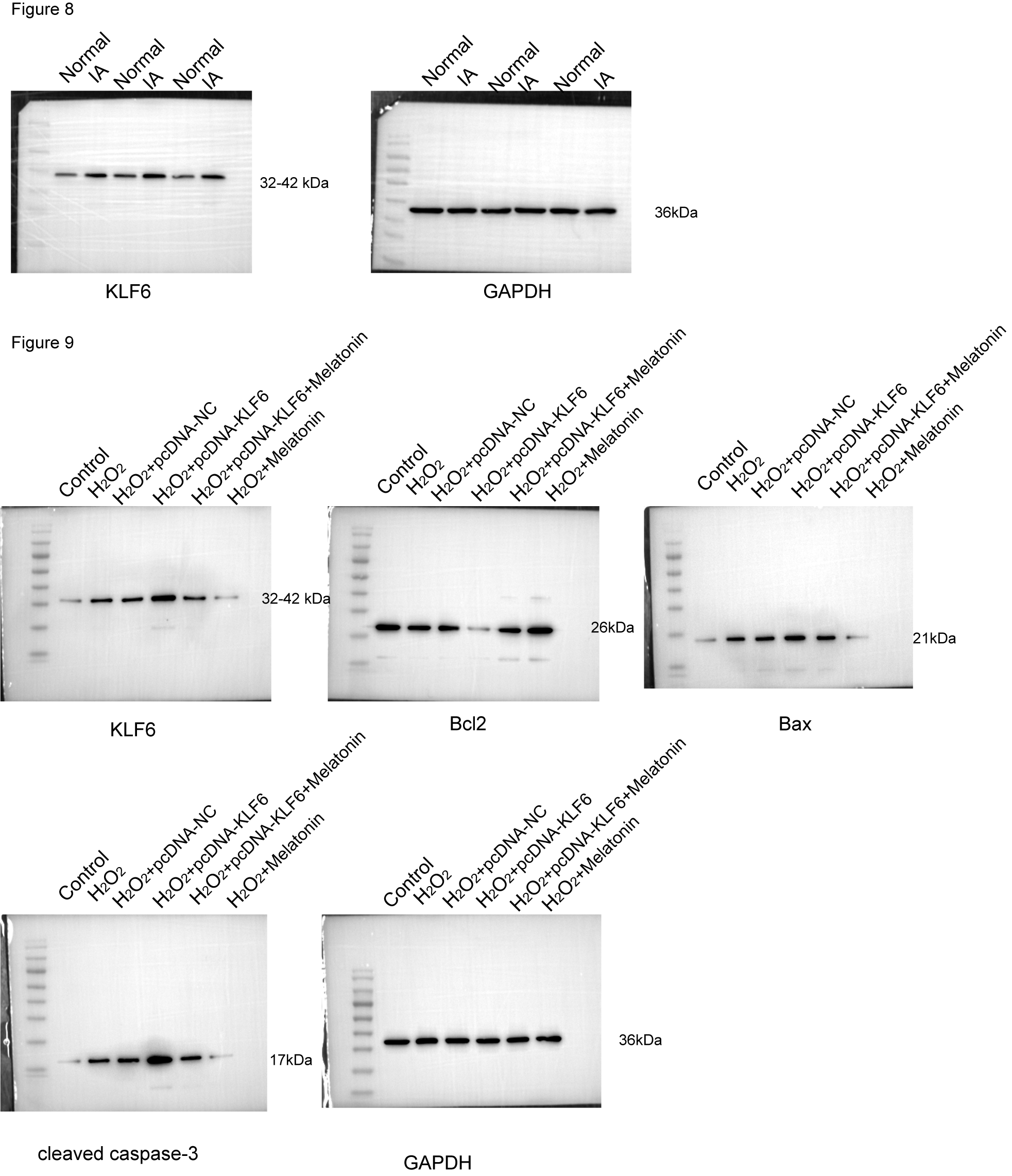

Supplement: Supplemental Material [file IANN_A_2397568_SM2661.zip › suppl_data/WB_original blot.tif]
